# Contemporary concepts in ‘exercise as medicine’ and related fields

**DOI:** 10.1113/EP093577

**Published:** 2026-03-24

**Authors:** Daniel H. Craighead, Casper Simonsen, Grit Elster Legaard, Cody G. Durrer, Helga Ellingsgaard, Andre Nyberg, Jill N. Barnes, Colleen S. Deane, Harry B. Rossiter, Damian M. Bailey, Bente Klarlund Pedersen, Ronan M. G. Berg

**Affiliations:** ^1^ School of Kinesiology University of Minnesota Minneapolis Minnesota USA; ^2^ Centre for Physical Activity Research Copenhagen University Hospital–Rigshospitalet Copenhagen Denmark; ^3^ Department of Community Medicine and Rehabilitation Umeå University Umeå Sweden; ^4^ Bruno Balke Biodynamics Laboratory University of Wisconsin‐Madison Madison Wisconsin USA; ^5^ School of Human Development and Health, Faculty of Medicine, Southampton General Hospital University of Southampton Southampton UK; ^6^ Respiratory Research Center The Lundquist Institute for Biomedical Innovation at Harbor‐UCLA Medical Center Torrance California USA; ^7^ Division of Respiratory and Critical Care Physiology and Medicine, Department of Medicine Harbor‐UCLA Medical Center Torrance California USA; ^8^ Neurovascular Research Laboratory, Faculty of Life Sciences and Education University of South Wales Pontypridd UK; ^9^ Department of Clinical Physiology and Nuclear Medicine Copenhagen University Hospital–Rigshospitalet Copenhagen Denmark; ^10^ Department of Clinical Medicine, Faculty of Health and Medical Sciences University of Copenhagen Copenhagen Denmark

## 1 INTRODUCTION

The field of translational exercise science faces the challenge of bridging mechanistic research and evidence‐based medicine (Berg et al., 2025). However, the field wrestles with the inconsistent use and definition of key terms, such as ‘physical activity’, ‘exercise’ or ‘intensity’, creating barriers to translation from mechanistic insight to clinical and public health application. This challenge highlights the importance of ongoing efforts to standardize definitions and terms in this field (Merrell et al., 2024). Forty years ago, Carl J. Caspersen and colleagues from the Centers for Disease Control and Prevention provided definitions for ‘physical activity’, ‘exercise’ and ‘physical fitness’ (Caspersen et al., 1985), which provided an interpretative framework that empowered research to relate these concepts to health. This work has had an enduring influence and accumulated >5000 citations (Web of Science; 1 December 2025). More recently, attempts have been made to standardize definitions of exercise intensity (Bishop et al., 2025; Norton et al., 2010) and (re)define terms such as durability, fatigability, repeatability and physiological resilience (Eldadah, 2010; Jones, 2024; Meixner et al., 2025). Such efforts are crucial for the aim of providing clinically relevant and evidence‐based exercise recommendations and prescriptions. With the continued need to revisit and reconsider key definitions, and in acknowledgement of the legacy of their seminal work, here we discuss the definitions of Caspersen et al. (1985) and offer complementary perspective for related terms that are frequently misunderstood or used interchangeably, such as ‘physical inactivity’ and ‘sedentary behaviour’ (Box 1).

**Box 1. Proposed definitions and updates**

Acute exercise bout: A single session of exercise, at a given duration and intensity, and that may include resistance or endurance exercise.

Balance: The maintenance of equilibrium while stationary or moving (Caspersen et al., 1985).

Body composition: The relative amounts of muscle, fat, bone and other vital parts of the body (Caspersen et al., 1985).

Cardiorespiratory endurance: Synonymous with terms such as cardiorespiratory fitness or aerobic capacity, cardiorespiratory endurance is defined by the body's maximal rate of oxygen transport and utilization.

Chronic exercise: Structured and repetitive acute exercise bouts, performed a specified number of times and at a defined frequency, leading to health benefits associated with physical fitness.

Exercise: A subset of physical activity that is planned and structured to achieve a final or intermediate objective of gaining health benefits associated with improving or maintaining physical fitness.

Exercise training: A subset of chronic exercise that is planned for the purpose of improving a specific outcome and that results in health‐related improvements in physical fitness.

Flexibility: The range of motion available at a joint (Caspersen et al., 1985).

Muscle strength: The amount of external force that a muscle can exert (Caspersen et al., 1985).

Muscular endurance: The ability of the muscle groups to exert external force for many repetitions or successive exertions in dynamic or static conditions that are not limited by the cardiovascular or pulmonary systems.

Physical activity: A quantifiable term referring to any bodily movement produced by the voluntary contraction of skeletal muscle that increases energy expenditure. This can encompass exercise in addition to occupational, domestic and all other activities.

Physical fitness: The anatomical and physiological qualities that provide an individual with the ability to meet the demands of a particular task in a steady state.

Physical inactivity: Physical activity levels below those required for optimal health and the prevention of premature death.

Power: The maximum rate at which one can perform work for no longer than a few seconds.

Sedentarism: Any waking behaviour characterized by an energy expenditure of 1.5 metabolic equivalents or lower.

## 2 PHYSICAL FITNESS

**FIGURE 1:**
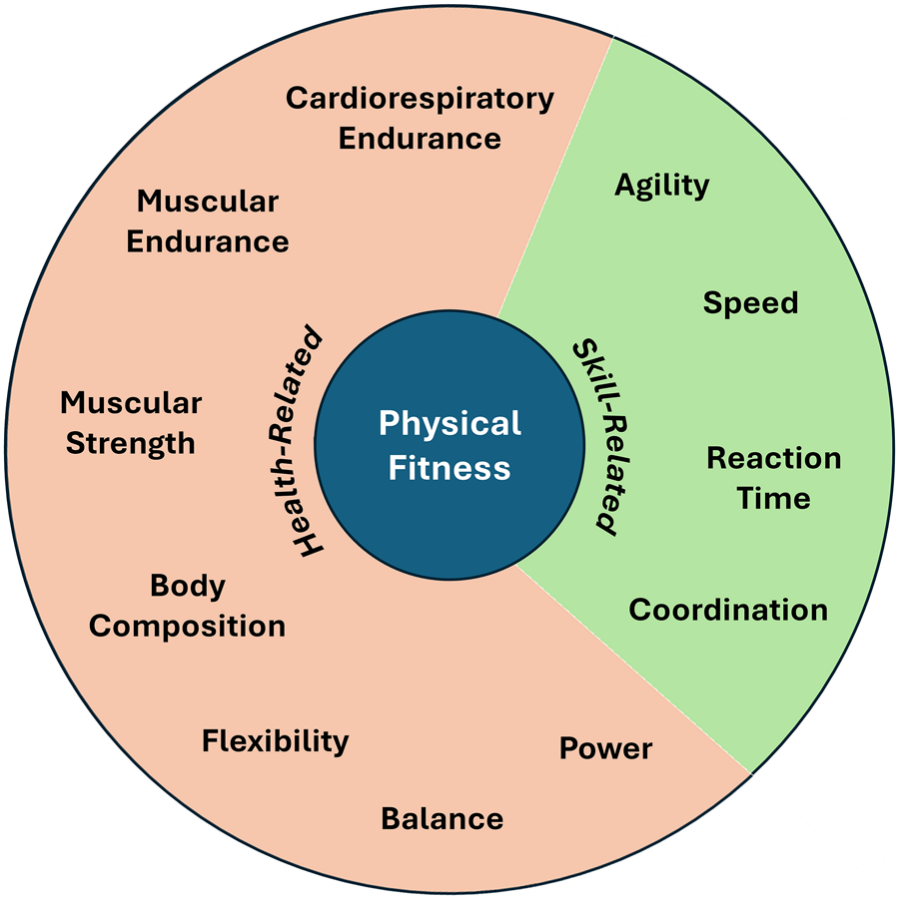
Health‐related and skill‐related components of physical fitness.

Caspersen et al. (1985) defined physical fitness as ‘a set of attributes that are either health‐ or skill‐related’. ‘Health‐related’ attributes were defined as: (1) cardiorespiratory endurance; (2) muscular endurance; (3) muscular strength; (4) body composition; and (5) flexibility. This definition is useful because the degree to which an individual possesses a particular attribute is quantifiable and can be measured using specific laboratory assessments (Table 1). These attributes have consistently shown strong associations with mortality, disease‐free survival and independent living (Araújo et al., 2024; Jochem et al., 2019; Kokkinos et al., 2023; Li et al., 2023; Roshanravan et al., 2017). In contrast, skill‐related domains of physical fitness were defined as: (1) agility; (2) balance; (3) coordination; (4) speed; (5) power; and (6) reaction time (Caspersen et al., 1985). As noted by Caspersen et al., health‐related components are more strongly related to health outcomes than are the skill‐related components. However, the definition by Caspersen et al. (1985) is also a limited one, because it constrains fitness attributes to five health‐related domains. With our ever‐increasing ability to assess specific physical fitness attributes and the relationship of these attributes to health, we are beginning to understand that some of the so‐called ‘skill‐related’ domains of physical fitness, such as power and balance, also play important roles in individual health. Therefore, a contemporary view of ‘exercise as medicine’ might benefit from a broader definition of physical fitness. In particular, both power and balance are strongly associated with risk of mortality (Araújo et al., 2025; Cao et al., 2021). In addition, these attributes are quantifiable (Table 1); thus, we propose that power and balance be included as health‐related attributes as we consider a broader definition of physical fitness (Figure 1).

**TABLE 1:** Techniques to assess the health‐related components of physical fitness.

Fitness component	Assessment technique
Cardiorespiratory endurance	Cardiorespiratory fitness, or aerobic capacity, is commonly evaluated by measuring symptom‐limited peak or maximum oxygen uptake during a cardiopulmonary exercise test, which involves incremental loading to the limit of tolerance (Wasserman & Whipp, 1975). Endurance capacity, or exercise tolerance, can be assessed by time trials or constant work rate endurance time; in the clinical setting, constant work rate endurance time has proved more sensitive than maximum oxygen uptake to quantify the efficacy of interventions (Casaburi et al., 2024; Puente‐Maestu et al., 2016). Critical power is the demarcation between the greatest metabolic rate for which oxygen uptake and blood lactate can stabilize and the lowest metabolic rate at which stabilization is not possible; thereby, quantifying the fitness definition of ‘the ability to meet the demands of a particular physical task in a steady state’ (Poole et al., 2021). However, accurate critical power assessment is highly demanding and rarely measured outside of the research setting. Submaximal assessments, such as the Åstrand–Ryhming cycle ergometer test (Cink & Thomas, 1981), the gas exchange or lactate threshold (Poole et al., 2021), or the measurement of oxygen uptake kinetics during constant work rate exercise (Poole & Jones, 2012; Rossiter, 2011), also provide information about cardiorespiratory fitness.
Muscular endurance	Can be assessed with static and dynamic tests, with the goal of assessing the duration or number of repetitions at which submaximal force can be produced. As an example, dynamic muscular endurance can be determined as the reduction in ability to produce muscular force during repeated isokinetic maximal contractions on a static dynamometer in which the speed of the movement is constant (Larsson & Karlsson, 1978). However, given that this measure relies on maximal rather than submaximal contractions, it might be a suboptimal indicator of muscular endurance. An alternative approach is to use repeated submaximal isotonic contractions in which the resistance applied to the muscle is fixed; for example, performing the test at a specified percentage of the one‐repetition maximum with a controlled repetition frequency and recording the time to exhaustion (Ozemak et al., 2025). Dynamic muscle endurance also can be assessed with tests that are easy to administer, such as the number of push‐ups, sit‐ups or squats that can be performed in 60 s (Vaara et al., 2012). Isometric muscular endurance is assessed as the time at which a submaximal contraction at a specified intensity can be maintained using an isometric dynamometer (Larsson & Karlsson, 1978).
Muscular strength	Often measured using one‐repetition maximum testing, which determines the maximum weight an individual can lift through its complete range of motion in a single repetition. Static muscle strength can be assessed as the maximal voluntary isometric contraction (e.g., using an isometric dynamometer), which can also be used to evaluate rate of force development and force impulse as indicators of explosive muscle strength without the confounding influence of joint angles (Maffiuletti et al., 2016). Handgrip dynamometry is another method used to assess isometric grip strength (Roberts et al., 2011).
Body composition	Dual energy X‐ray absorptiometry allows for measurement of whole‐body and regional bone mineral density, lean mass and fat mass (Laskey, 1996). MRI and CT allow for regional assessment of fat and lean mass (Borga, 2018; Niklasson et al., 2022). Hydrostatic weighing and whole‐body air displacement plethysmography are additional powerful techniques to assess fat and lean mass based on estimating density, although these outcomes are limited to whole‐body (rather than regional) measurements (Fields et al., 2002; Timson & Coffman, 1984). Bioelectrical impedance analysis represents a commercially available technique to assess body composition, although the accuracy varies across devices (Siedler et al., 2023). Skinfold callipers can be used at multiple sites across the body and purport to measure the percentage body fat, although it is important to note that the callipers measure only subcutaneous, and not visceral, fat (Gomes et al., 2020).
Flexibility	Can be measured via goniometry, which involves measuring joint angles at their maximum range of motion. Field tests, such as the sit‐and‐reach test, which have normative values, assess flexibility of isolated body segments (Committee on Fitness Measures and Health Outcomes in Youth; Food and Nutrition Board; Institute of Medicine, 2012). The sit‐and‐reach test, for example, measures flexibility of the hamstrings and lower back.
Balance	Most often assessed by asking participants to maintain increasingly challenging postural positions and providing a score based on the point at which participants can no longer maintain the position without correction. For example, the balance test component of the short physical performance battery asks participants to stand with their feet side by side, semi‐tandem and full tandem for 10 s each (Eusepi et al., 2025). Examples of other tests that use unique protocols with the same overall principle of progressing difficulty are the modified Romberg test (Agrawal et al., 2011) and the balance error scoring system (Riemann et al., 1999).
Power	Can be assessed using non‐complex movements, such as a leg press or knee extension, on appropriately instrumented weight machines. Resistance is set to a prespecified portion of the participant's one‐repetition maximum, and participants then perform the concentric component on the lift as quickly as possible to produce peak power (Parrino et al., 2024; Petrella et al., 2005). Very short (3–4 s) bouts of maximal exercise on a cycle ergometer also provide a way to assess power (Ferguson et al., 2025; Martin et al., 1997). Can also be assessed in the field without specialized equipment; an example of this is the sit‐to‐stand test, which measures how quickly participants can rise from a chair five times (Alcazar et al., 2018).

The term ‘fitness’ has been used from at least the 16th century. Before Darwin used it to describe the ‘quality of fulfilling the requirements of a particular environment for survival and reproduction’, the biological definition of physical fitness was the ‘ability to fulfil a particular physical task’ (see Box 2 regarding task specificity). As such, fitness encompasses a broad definition of anatomical and physiological attributes. Furthermore, outside of athletic training and competitions, humans rarely perform tasks in our daily lives that challenge the maximum capacity for these attributes. Therefore, a more contemporary definition of physical fitness, particularly in the context of ‘exercise as medicine’, might be ‘the ability to meet the demands of a particular physical task in a steady state’ (Sietsema et al., 2021). This recognizes that the relationship between maximum capacity of the neuromuscular–cardiorespiratory systems to power exercise and the ability to sustain exercise at high fractions of that maximum power are both malleable traits. Given that the mechanism(s) that link physical fitness to health remain elusive, the lexicon of attributes that contribute to physical fitness should be broad enough to facilitate their discovery.

**Box 2. Sir Steven Redgrave and task specificity**

Soon after the British rower Sir Steven Redgrave won his fifth consecutive Olympic gold medal in 2000, he ran the London Marathon. With 16 years as the world's top rower, few would deny that Sir Redgrave was among the fittest endurance athletes in history. However, even with a maximum oxygen uptake approaching ∼7 L/min, he was unable to lug his 108 kg and 195 cm frame over 42.2 km in a time better than 4 h 55 min; far off times achieved by elite marathon runners (Maffetone et al., 2017). In contrast, elite runners, who tend to be shorter and weigh less than Sir Redgrave, may be capable of sustaining an effort equivalent to 90% of their maximum oxygen uptake during an entire race (Jones et al., 2021), contributing to their elite times. This example emphasizes the task specificity of physical fitness.

With the above in mind, we will briefly revisit the definition of each health‐related component of physical fitness. The definition of cardiorespiratory endurance offered by Caspersen et al. (1985) has some shortcomings. The term cardiorespiratory endurance was envisioned as synonymous with more contemporary terms, such as ‘cardiorespiratory fitness’ or ‘aerobic capacity’, and is a prime mediator of the ability to sustain endurance exercise. We propose that cardiorespiratory endurance should be defined by the body's ‘maximum rate of oxygen transport and utilization’, because it depends on the effective functioning of the pulmonary, circulatory, metabolic and neuromuscular systems to supply substrates (particularly oxygen) required for repeated muscle contractions and eliminate products of metabolism that contribute to fatigue, dyspnoea or pain. Assessment techniques for cardiorespiratory endurance therefore require sufficient challenge to all the physiological systems involved, necessitating sustained activation of a large muscle mass, high rates of convective and diffusive oxygen transport and high rates of ventilation that are needed to achieve maximum oxygen uptake (Table 1). Importantly, cardiorespiratory endurance is very strongly related to all‐cause mortality (Kokkinos et al., 2023; Mandsager et al., 2018), supporting its status as a pillar of good health.

Caspersen et al. (1985) defined muscular endurance as ‘the ability of the muscle groups to exert external force for many repetitions or successive exertions’. However, a challenge associated with this definition is that repetitive muscle contractions also tend to occur in activities that test cardiorespiratory endurance. Therefore, it seems appropriate to update the definition of muscular endurance to ‘the ability of the muscle groups to exert external force for many repetitions or successive exertions in dynamic or static conditions that are not limited by the cardiovascular or pulmonary systems’. This definition more closely aligns with the small muscle mass protocols used to assess muscular endurance (Table 1). This update will also help to differentiate cardiorespiratory endurance from muscular endurance. Overall, muscular endurance is an important health‐related component of physical fitness, because it is associated with mortality and physical function (Roshanravan et al., 2017).

Muscle strength is appropriately defined by Caspersen et al. (1985) as ‘the amount of external force that a muscle can exert’. Muscle strength is associated with all‐cause mortality in both healthy and patient populations (García‐Hermoso et al., 2018; Jochem et al., 2019). It is important to acknowledge that muscular strength is associated with muscle mass and, more specifically, physiological cross‐sectional area (Riviati & Indra, 2023), a component of body composition, which again highlights the interplay between health‐related components of physical fitness.

Body composition is ‘the relative amounts of muscle, fat, bone, and other vital parts of the body’, while flexibility is ‘the range of motion available at a joint’ (Caspersen et al., 1985). As with the other components discussed, these factors contribute to individual health. Indeed, measures of both body composition and flexibility are related to all‐cause mortality (Araújo et al., 2024; Li et al., 2023).

As stated above, we propose that balance be reclassified from a skill‐related to a health‐related component of physical fitness. This is because balance disorders are associated with increased all‐cause mortality, in addition to increased risk of death from cardiovascular diseases and cancer (Cao et al., 2021). Beyond this reclassification, the definition of balance as ‘the maintenance of equilibrium while stationary or moving’ (Caspersen et al., 1985) remains appropriate. Power is the other component of physical fitness that we propose should fall under health‐related rather than skill‐related attributes. It is now known that power, defined as ‘the rate at which one can perform work’, is strongly related to mortality (Alcazar et al., 2021; Araújo et al., 2025) and is therefore very important for maximizing individual health. This definition is somewhat ambiguous because the rate at which one can perform work depends strongly on the duration for which that power is measured. For example, aerobic power might be defined as the power output that can be supported at maximum oxygen uptake. However, the definition here relates to peak power output, which should be assessed over short intervals of time. We therefore propose that power be defined as ‘the maximum rate at which one can perform work for no longer than a few seconds’. Overall, understanding the health‐related components of physical fitness provides important insight into how to use exercise as medicine to improve health and well‐being.

## 3 PHYSICAL ACTIVITY

Caspersen et al. (1985) define ‘physical activity’ as any bodily movement produced by skeletal muscle contraction that results in energy expenditure. They categorize physical activity in daily life into occupational, sports, conditioning, household or other activities. Like physical fitness, physical activity is a quantifiable entity. As specified by Caspersen et al., the increase in energy expenditure attributable to physical activity can be assessed over a specified duration. However, accurate assessment of daily expenditure is complex. Indirect calorimetry requires equipment to measure oxygen uptake and carbon dioxide output and is typically applied during individual physical tasks rather than for extended durations (Macfarlane, 2017; Overstreet et al., 2017). The doubly labelled water method is the gold standard for measuring total daily energy expenditure in free‐living humans (Buchowski, 2014) but is expensive to apply in large cohorts. Historically, researchers have relied on questionnaires and, more recently, on accelerometery to estimate energy expenditure. These estimates, which suffer from variability and inaccuracy (Plasqui & Westerterp, 2007; Prince et al., 2008), are based on the concept of metabolic equivalents (METs). METs are the ratio of energy expenditure, relative to body mass, during daily living compared with a reference, which is, by convention, fixed for all people at 3.5 mL of oxygen consumption per kilogram of body mass per minute (this value represents the mean resting energy expenditure during quiet sitting of an average person). One MET is also equal to 1 kcal/kg body mass/h. The use of counting ‘steps’ with pedometers as a measure of physical activity is not recommended, because steps do not capture energy expenditure directly (Kumahara et al., 2009; Nielson et al., 2011).

Energy expenditure measured via doubly labelled water, accelerometers and questionnaires is associated with important health outcomes (Andersen et al., 2014; Dempsey et al., 2022; Manini et al., 2006). Current physical activity guidelines are based on these observational studies that associate mortality risk with physical activity and call for accumulating ≥150 min of moderate‐intensity (defined as activity that requires 3–6 METs) or 75 min of vigorous‐intensity (> 6 METs) physical activity (or a combination) per week (World Health Organization, 2021). Unfortunately, we are lacking randomized controlled trials that are of long enough duration and adequately powered to assess the effects of physical activity on survival. The ‘Generation 100’ study (Stensvold et al., 2015) is an example of a randomized controlled trial to examine the effect of a 5 year exercise training programme on survival in >1500 people >70 years of age. Although there was no difference in mortality in the control, moderate‐ or high‐intensity training groups, the overall mortality rate in the study was low, limiting the conclusions of the study. As yet, a causal link remains elusive. Additionally, randomized controlled trials on the effect of high rates of physical activity during early life or midlife on survival have yet to be performed. An important caveat to this story is whether it is physical activity (energy expenditure) or physical fitness (the ability to meet a particular task in a steady state) that imparts health and survival benefit (e.g., Myers et al., 2021). High rates of energy expenditure confer beneficial physiological adaptations when they are frequent, intense and progressive, and subsequent adaptations are specific to the tissues and energy systems involved. Hence, the ‘one‐size‐fits‐all’ MET framework might obfuscate the causal relationships that studies are attempting to elucidate. Nonetheless, physical activity is a central tenet of the ‘exercise as medicine’ framework.

## 4 ACUTE VERSUS CHRONIC EXERCISE

In the paper by Caspersen et al. (1985), ‘exercise’ is defined as a subset of physical activity that is planned, structured and repetitive, with a final or intermediate objective of improving or maintaining physical fitness. Although valuable, we believe that this definition requires some qualification. In our experience, this definition does not adequately define a single acute bout of exercise, which might be spontaneous, might not be repeated and might not have the final or immediate objective of improving or maintaining physical fitness. An ‘acute exercise bout’ refers to a single session of exercise, defined by a given duration and intensity, and that might include resistance or endurance exercise. Chronic exercise, in contrast, is ‘structured and repetitive’. ‘Structured’ means that the exercise has a defined duration, intensity and type, whereas ‘repetitive’ denotes the frequency of acute structured exercise bouts. Chronic exercise encompasses ‘exercise training’, which includes chronic exercise performed with the objective of achieving a specific goal. In the spirit of this ‘exercise as medicine’ article collection, we propose that the concept of exercise training should include gaining health benefits associated with physical fitness. This is not to exclude improved physical performance (e.g., winning in a sports competition or improving a personal best); however, from a public health standpoint we believe it is important to identify the link between exercise and health; a link that is mediated (in still emerging ways) by physical fitness. We therefore advocate for the use of the terms ‘acute exercise’ and ‘chronic exercise’ (or ‘exercise training’ when appropriate) to distinguish between a ‘single bout’ of exercise and exercise that is ‘structured and repetitive’, where exercise itself is defined as ‘a subset of physical activity that is planned and structured to achieve the final or intermediate objective of gaining health benefits associated with improving or maintaining physical fitness’.

## 5 PHYSICAL INACTIVITY VERSUS SEDENTARISM

Caspersen et al. (1985) do not provide definitions for the terms ‘physical inactivity’ and ‘sedentary behaviour’. Both terms should be distinguished from ‘rest’, which can be expressed as either basal metabolic rate or resting metabolic rate. Basal metabolic rate has classically been defined as a condition where all skeletal muscles, apart from those involved in normal tidal breathing, are inactive, with no motor impulses from the CNS, and is the minimum rate of energy expenditure needed for life (Asmussen, 1967; Henry, 2005). Resting metabolic rate, in contrast, is measured in somewhat less stringent conditions (e.g., after only a 15 min period of seated rest; McMurray et al., 2014) and therefore will be slightly higher than basal metabolic rate (Levine, 2005). Although the Centers for Disease Control and Prevention definitions for exercise do not encompass ‘physical inactivity’, the World Health Organization uses the term to describe the failure to meet current physical activity recommendations (World Health Organization, 2021). Although this definition is particularly useful in public health research and recommendations, it might be overly rigid when applied to mechanistic studies and clinical trials in exercise physiology. It might also underestimate how ‘active’ someone is. We propose the adoption of a more flexible definition, such as that formulated by Frank Booth and colleagues (Booth et al., 2012), who defined physical inactivity as ‘physical activity levels below those required for optimal health and the prevention of premature death’. This conceptual definition should remain relevant within an individual's evolving physical and dietary environment and offers a more personalized perspective. Although we recognize its potential limitations (i.e., that it might be considered vague in absolute terms and difficult to operationalize in practical contexts), it nevertheless provides a framework that acknowledges personalized approaches to medicine.

Importantly, sedentarism is distinct from physical inactivity according to this definition. Instead, it has previously been defined as any waking behaviour characterized by an energy expenditure of ≤1.5 METs while sitting, reclining or lying (Sedentary Behavior Research Network, 2012). Examples of sedentary behaviours include most desk‐based office work, driving and watching television. The WHO guidelines further operationalize this definition to include self‐reported low‐movement sitting activities across leisure time, occupational settings and total sitting time, including screen‐related activities, such as watching television (World Health Organization, 2021). Although the 1.5 MET threshold seems reasonable, some sitting behaviours, such as typing, can exceed this threshold, whereas standing still can be <1.5 METs in certain individuals (Mansoubi et al., 2015). As such, this threshold‐specific definition should always be considered in the context of the specific research question and study objectives. Notably, although there is a threshold at which someone could be classified as physically active or inactive, sedentarism is more likely to be quantified as a continuous variable measured through devices, such as accelerometers and inclinometers, in addition to questionnaires and 24 h recall methods (Aunger & Wagnild, 2022). Of note, inactive individuals are likely to spend a considerable amount of time being sedentary, but even those who are physically active, including elite athletes, spend periods of time performing sedentary behaviour.

## 6 CONCLUDING REMARKS

As in any other domain of science, field‐specific definitions of central terms are essential within ‘exercise as medicine’ and related fields. To remain relevant and operational, some definitions must evolve clearly and adapt as the field progresses. The updated definitions proposed in Box 1 represent a humble attempt to achieve exactly that.

## AUTHOR CONTRIBUTIONS

All authors were involved in the conception and design of the work, and all authors contributed to drafting the work or revising it critically for important intellectual content. All authors approved the final version of the manuscript and agree to be accountable for all aspects of the work in ensuring that questions related to the accuracy or integrity of any part of the work are appropriately investigated and resolved. All persons designated as authors qualify for authorship, and all those who qualify for authorship are listed.

## CONFLICT OF INTEREST

Harry Rossiter reports consulting fees from the NIH RECOVER‐ENERGIZE working group (1OT2HL156812) and is involved in contracted clinical research with GlaxoSmithKline, Genentech, Intervene Immune, Mezzion, Regeneron, Respira, Roche, Biocient and United Therapeutics. He is a visiting Professor at the University of Leeds, UK and the University of Pavia, Italy. He reports a pending patent application filed by The Lundquist Institute, titled ‘Testing System to Diagnose Neuromuscular Deconditioning and Pathologic Conditions’. Damian Bailey is Editor‐in‐Chief of *Experimental Physiology*, Chair of the Life Sciences Working Group, a member of the Human Spaceflight and Exploration Science Advisory Committee to the European Space Agency and a member of the Space Exploration Advisory Committees to the UK and Swedish Space Agencies. Damian Bailey is also a member of the National Cardiovascular Network for Wales and South‐East Wales Vascular Network. André Nyberg reports consultant fees from AstraZeneca and Chiesi.

## FUNDING INFORMATION

The Centre for Physical Activity Research (CFAS) is supported by TrygFonden (grants ID 101390, ID 20045, ID 125132 and ID 177225). The funders had no role in study design, data collection and analysis, decision to publish or preparation of the manuscript. Harry Rossiter is supported by grants from National Institutes of Health (R01HL151452, R01HL166850, R01HL153460, P50HD098593 and R01DK122767), Tobacco Related Disease Research Program (T31IP1666) and Department of Defense/USAMRAA (HT9425‐24‐1‐0249). André Nyberg is supported by ongoing grants from the European Research Council (101078602), the Swedish Research Council (20230341), the Swedish Heart and Lung Foundation (2023034 and 20250454), the Strategic Research Area Health Care Science and Umeå University. Damian Bailey is a Royal Society Wolfson Research Fellow (WM17007).
